# Supplementary material for: Somno-Art Software identifies pathology-induced changes in sleep parameters similarly to polysomnography
Source: PLoS One. 2023 Oct 20;18(10):e0291593. doi: 10.1371/journal.pone.0291593 (PMC10588897; doi:10.1371/journal.pone.0291593)
Supplement: S1 Dataset — (PDF) [file pone.0291593.s002.pdf]

Patho	Subj	PSG_TST	SA_TST	PSG_SL	SA_SL	PSG_WASO	SA_WASO	PSG_SE	SA_SE	PSG_REML	SA_REML	PSG_N1N2	SA_N1N2	PSG_N3	SA_N3	PSG_REM	SA_REM	PSG_NREM	SA_NREM
INS	1	279.17	266.83	51.33	55.33	58.00	66.33	71.86	68.68	126.67	84.00	133.50	115.50	88.67	81.33	57.00	70.00	222.17	196.83
INS	2	198.00	199.25	95.25	95.00	95.25	94.25	50.97	51.29	57.25	134.25	109.00	124.25	63.75	53.50	25.25	21.50	172.75	177.75
INS	3	174.33	259.67	36.67	53.17	177.50	75.67	44.87	66.84	NaN	196.33	123.83	175.83	33.17	43.33	17.33	40.50	157.00	219.17
INS	4	221.83	221.83	74.17	78.17	92.50	88.50	57.10	57.10	42.83	81.33	177.17	138.50	6.50	44.67	38.17	38.67	183.67	183.17
INS	5	255.25	277.50	61.25	18.50	72.00	92.50	65.70	71.43	NaN	54.00	197.50	200.50	36.25	48.50	21.50	28.50	233.75	249.00
INS	6	210.25	248.25	81.00	79.50	97.25	60.75	54.12	63.90	76.00	78.25	105.50	180.00	72.75	34.50	32.00	33.75	178.25	214.50
INS	7	209.00	190.25	94.50	109.75	85.00	88.50	53.80	48.97	143.50	214.25	132.50	109.00	37.25	64.00	39.25	17.25	169.75	173.00
INS	8	333.50	327.50	26.00	25.50	29.00	35.50	85.84	84.30	63.75	72.75	219.00	186.75	44.25	45.00	70.25	95.75	263.25	231.75
INS	9	250.00	302.00	77.25	36.50	61.25	50.00	64.35	77.73	16.25	57.00	125.00	153.25	48.75	82.50	76.25	66.25	173.75	235.75
INS	10	248.00	224.00	118.00	121.00	22.50	43.50	63.84	57.66	98.00	90.00	130.00	103.50	53.00	54.50	65.00	66.00	183.00	158.00
INS	11	262.83	254.67	53.17	67.33	72.50	66.50	67.65	65.55	81.83	69.50	173.50	142.83	27.00	55.33	62.33	56.50	200.50	198.17
INS	12	274.00	295.00	30.50	26.50	84.00	67.00	70.53	75.93	87.25	126.25	169.50	205.75	56.75	43.00	47.75	46.25	226.25	248.75
INS	13	345.00	346.50	10.00	6.50	33.50	35.50	88.80	89.19	57.50	56.50	147.00	175.50	130.00	93.00	68.00	78.00	277.00	268.50
INS	14	299.25	326.00	21.00	10.50	68.25	52.00	77.03	83.91	181.25	92.50	143.50	132.50	107.75	123.75	48.00	69.75	251.25	256.25
INS	15	360.00	352.25	19.25	20.00	9.25	16.25	92.66	90.67	51.00	50.75	169.50	196.00	114.25	83.50	76.25	72.75	283.75	279.50
INS	16	214.75	301.75	119.75	74.50	54.00	12.25	55.28	77.67	53.00	63.75	79.75	234.50	75.00	20.50	60.00	46.75	154.75	255.00
INS	17	362.50	344.00	10.50	21.50	15.50	23.00	93.31	88.55	83.50	59.00	144.50	151.00	123.25	109.25	94.75	83.75	267.75	260.25
INS	18	337.00	285.00	35.00	52.50	16.50	51.00	86.74	73.36	64.50	50.50	183.00	174.00	82.00	45.00	72.00	66.00	265.00	219.00
INS	19	331.50	290.00	37.00	42.50	20.00	56.00	85.33	74.65	131.50	125.00	145.50	123.00	124.00	114.50	62.00	52.50	269.50	237.50
INS	20	294.50	253.67	43.83	48.33	50.17	86.50	75.80	65.29	93.67	89.17	118.00	137.33	123.83	60.33	52.67	56.00	241.83	197.67
INS	21	358.00	329.00	7.00	6.50	23.50	53.00	92.15	84.68	135.00	132.50	273.50	214.00	26.00	58.50	58.50	56.50	299.50	272.50
INS	22	352.00	364.50	17.00	14.50	19.50	9.50	90.60	93.82	13.00	116.00	186.00	197.50	89.00	87.00	77.00	80.00	275.00	284.50
INS	23	353.00	350.50	15.00	24.50	20.50	13.50	90.86	90.22	11.50	74.00	216.00	251.00	36.00	50.50	101.00	49.00	252.00	301.50
INS	24	353.00	347.00	10.75	11.25	24.75	30.25	90.86	89.32	76.50	73.50	197.25	164.25	62.75	72.25	93.00	110.50	260.00	236.50
INS	25	330.00	305.75	20.25	39.00	38.25	43.75	84.94	78.70	57.50	80.00	121.00	171.25	136.75	65.50	72.25	69.00	257.75	236.75
INS	26	324.00	301.50	14.50	15.50	50.00	71.50	83.40	77.61	58.50	49.00	158.00	204.00	123.00	51.00	43.00	46.50	281.00	255.00
INS	27	275.50	304.25	83.00	38.75	30.00	45.50	70.91	78.31	74.75	117.75	130.75	185.75	88.25	57.00	56.50	61.50	219.00	242.75
INS	28	341.00	329.25	22.00	22.50	25.50	36.75	87.77	84.75	58.50	57.50	156.25	234.75	127.50	52.50	57.25	42.00	283.75	287.25
INS	29	355.50	321.00	7.00	6.00	26.00	61.50	91.51	82.63	117.50	155.50	212.00	184.50	68.50	103.00	75.00	33.50	280.50	287.50
INS	30	303.75	298.75	29.00	9.25	55.75	80.50	78.19	76.90	103.25	84.75	159.50	205.25	87.50	58.75	56.75	34.75	247.00	264.00
INS	31	182.67	249.50	182.00	45.67	23.83	93.33	47.02	64.22	58.17	195.17	77.00	131.67	60.50	77.33	45.17	40.50	137.50	209.00

INS	32	317.25	342.50	26.25	20.25	45.00	25.75	81.66	88.16	105.50	110.75	144.50	192.75	99.75	89.50	73.00	60.25	244.25	282.25
INS	33	289.67	300.33	83.83	72.67	15.00	15.50	74.56	77.31	90.83	100.50	91.00	161.17	147.83	64.50	50.83	74.67	238.83	225.67
INS	34	256.75	256.75	113.50	91.50	18.25	40.25	66.09	66.09	105.25	121.25	140.00	167.50	73.75	63.25	43.00	26.00	213.75	230.75
INS	35	349.00	368.00	19.50	5.00	20.00	15.50	89.83	94.72	82.50	62.00	161.50	237.00	138.50	41.00	49.00	90.00	300.00	278.00
INS	36	236.50	222.50	62.50	81.33	89.50	84.67	60.88	57.27	209.17	190.50	190.33	123.00	7.67	64.67	38.50	34.83	198.00	187.67
INS	37	279.00	308.00	35.50	4.00	74.00	76.50	71.81	79.28	119.50	105.50	185.00	187.50	44.50	58.50	49.50	62.00	229.50	246.00
INS	38	311.50	317.67	28.50	23.17	48.50	47.67	80.18	81.77	101.50	125.33	165.67	165.00	88.67	91.00	57.17	61.67	254.33	256.00
INS	39	335.00	316.50	4.00	6.50	49.50	65.50	86.23	81.47	79.50	85.00	220.50	164.00	47.50	86.50	67.00	66.00	268.00	250.50
INS	40	338.75	354.00	16.75	5.75	33.00	28.75	87.19	91.12	100.25	97.00	184.75	229.50	71.00	65.00	83.00	59.50	255.75	294.50
INS	41	211.50	233.83	25.00	27.50	152.00	127.17	54.44	60.19	NaN	NaN	172.50	115.17	7.83	74.67	31.17	44.00	180.33	189.83
INS	42	264.17	281.67	24.83	34.67	99.50	72.17	68.00	72.50	118.17	55.00	202.67	204.67	26.17	38.83	35.33	38.17	228.83	243.50
INS	43	331.75	334.75	12.50	19.75	44.25	34.00	85.39	86.16	134.25	121.00	160.50	120.50	102.25	151.25	69.00	63.00	262.75	271.75
INS	44	280.83	273.83	28.83	34.33	78.83	80.33	72.29	70.48	70.33	63.83	147.83	115.67	62.83	78.33	70.17	79.83	210.67	194.00
INS	45	208.50	278.50	83.83	66.50	96.17	43.50	53.67	71.69	73.83	78.83	128.50	146.50	38.83	69.17	41.17	62.83	167.33	215.67
INS	46	378.00	366.00	6.50	5.00	4.00	17.50	97.30	94.21	65.50	144.00	197.50	172.50	88.50	133.00	92.00	60.50	286.00	305.50
INS	47	341.25	345.75	19.50	16.75	27.75	26.00	87.84	89.00	57.25	130.75	213.75	200.00	64.25	79.75	63.25	66.00	278.00	279.75
INS	48	291.50	321.50	34.25	33.75	62.75	33.25	75.03	82.75	119.50	57.50	196.00	164.75	55.25	87.75	40.25	69.00	251.25	252.50
INS	49	327.00	345.50	13.75	11.00	47.75	32.00	84.17	88.93	59.00	73.25	238.00	264.50	4.25	38.00	84.75	43.00	242.25	302.50
INS	50	319.00	350.50	14.00	8.00	55.50	30.00	82.11	90.22	114.00	53.50	165.00	227.50	94.50	82.50	59.50	40.50	259.50	310.00
INS	51	256.00	282.17	51.83	37.17	80.67	69.17	65.89	72.63	148.00	156.00	173.83	210.00	27.17	21.83	55.00	50.33	201.00	231.83
INS	52	328.00	317.50	15.50	15.00	45.00	56.00	84.43	81.72	59.50	62.00	226.00	207.50	35.00	31.50	67.00	78.50	261.00	239.00
INS	53	287.75	351.75	13.00	12.75	87.75	24.00	74.07	90.54	216.50	73.00	157.25	198.25	107.25	86.75	23.25	66.75	264.50	285.00
INS	54	363.00	349.50	9.50	18.50	16.00	20.50	93.44	89.96	177.00	77.00	142.50	169.00	158.00	110.50	62.50	70.00	300.50	279.50
INS	55	274.25	268.25	68.75	69.00	45.50	51.25	70.59	69.05	95.25	97.00	145.00	118.25	46.00	88.25	83.25	61.75	191.00	206.50
INS	56	218.50	241.75	105.25	106.00	64.75	40.75	56.24	62.23	82.00	84.50	102.00	115.25	92.75	100.00	23.75	26.50	194.75	215.25
INS	57	195.00	198.75	102.75	72.75	90.75	117.00	50.19	51.16	49.75	197.75	109.75	141.50	40.75	25.75	44.50	31.50	150.50	167.25
INS	58	209.83	205.33	61.00	68.33	117.67	114.83	54.01	52.85	114.50	58.50	110.83	106.17	46.00	48.50	53.00	50.67	156.83	154.67
INS	59	202.83	198.50	78.50	89.50	107.17	100.50	52.21	51.09	75.33	61.67	112.00	108.83	61.33	60.17	29.50	29.50	173.33	169.00
INS	60	341.17	341.17	23.00	21.17	24.33	26.17	87.82	87.82	99.00	78.00	214.83	193.17	64.17	76.00	62.17	72.00	279.00	269.17
INS	61	260.67	267.33	105.00	84.00	22.83	37.17	67.10	68.81	141.00	82.00	109.17	110.00	117.67	107.50	33.83	49.83	226.83	217.50
INS	62	284.67	278.00	47.83	68.00	56.00	42.50	73.27	71.56	95.33	74.50	200.33	151.67	43.50	76.00	40.83	50.33	243.83	227.67
INS	63	202.50	225.50	98.00	91.00	88.00	72.00	52.12	58.04	84.50	86.00	120.00	111.50	52.50	71.50	30.00	42.50	172.50	183.00
INS	64	229.50	238.25	71.25	72.00	87.75	78.25	59.07	61.33	96.25	69.25	109.75	117.50	86.75	59.25	33.00	61.50	196.50	176.75

INS	65	269.00	264.17	63.17	69.33	56.33	55.00	69.24	68.00	82.83	67.00	131.83	137.83	64.17	66.17	73.00	60.17	196.00	204.00
INS	66	318.00	269.00	21.50	30.75	49.00	88.75	81.85	69.24	154.75	156.75	240.75	178.50	40.75	58.50	36.50	32.00	281.50	237.00
MDD	1	367.25	343.75	14.75	15.25	6.50	29.50	94.53	88.48	57.50	56.75	189.50	170.50	96.75	92.50	81.00	80.75	286.25	263.00
MDD	2	355.00	319.50	19.50	35.25	14.00	33.75	91.38	82.24	60.50	41.50	163.75	192.25	133.25	71.75	58.00	55.50	297.00	264.00
MDD	3	333.75	323.00	34.50	35.75	20.25	29.75	85.91	83.14	78.75	75.25	183.75	180.25	77.25	73.25	72.75	69.50	261.00	253.50
MDD	4	304.00	274.25	31.00	67.00	53.50	47.25	78.25	70.59	140.25	46.50	158.00	151.50	111.75	66.50	34.25	56.25	269.75	218.00
MDD	5	375.00	347.00	8.00	8.50	5.50	33.00	96.53	89.32	44.00	40.50	168.00	195.00	130.00	46.50	77.00	105.50	298.00	241.50
MDD	6	313.00	313.25	33.75	35.00	41.75	40.25	80.57	80.63	171.75	169.25	180.75	151.50	94.75	126.25	37.50	35.50	275.50	277.75
MDD	7	352.25	347.50	21.75	24.25	14.50	16.75	90.67	89.45	76.75	125.50	129.75	179.75	150.75	90.50	71.75	77.25	280.50	270.25
MDD	8	291.25	352.75	16.75	5.50	80.50	30.25	74.97	90.80	134.00	90.75	165.50	225.25	82.00	69.25	43.75	58.25	247.50	294.50
MDD	9	351.00	328.00	10.50	29.50	27.00	31.00	90.35	84.43	16.50	99.50	173.00	255.50	124.00	29.50	54.00	43.00	297.00	285.00
MDD	10	250.50	298.25	87.75	23.50	50.25	66.75	64.48	76.77	116.00	118.00	157.25	187.00	14.75	46.75	78.50	64.50	172.00	233.75
MDD	11	310.75	297.25	50.25	25.25	27.50	66.00	79.99	76.51	96.50	89.00	117.00	201.00	131.75	42.00	62.00	54.25	248.75	243.00
MDD	12	340.00	301.50	24.25	23.50	24.25	63.50	87.52	77.61	213.50	175.25	181.50	151.25	118.25	113.25	40.25	37.00	299.75	264.50
MDD	13	304.50	303.50	24.00	2.00	60.00	83.00	78.38	78.12	139.00	169.00	220.00	182.00	39.50	64.50	45.00	57.00	259.50	246.50
MDD	14	314.50	347.00	20.00	12.75	54.00	28.75	80.95	89.32	209.00	146.25	229.50	222.75	58.25	48.00	26.75	76.25	287.75	270.75
MDD	15	1.25	1.75	NaN	NaN	NaN	NaN	0.32	0.45	NaN	NaN	1.25	1.75	0.00	0.00	0.00	0.00	1.25	1.75
MDD	16	346.50	276.00	18.00	50.50	24.00	62.00	89.19	71.04	150.50	114.00	95.00	163.50	196.00	60.00	55.50	52.50	291.00	223.50
MDD	17	331.25	330.00	12.00	8.50	45.25	50.00	85.26	84.94	113.25	114.25	210.75	178.00	41.75	69.25	78.75	82.75	252.50	247.25
MDD	18	342.25	342.00	8.25	33.00	38.00	13.50	88.10	88.03	232.50	85.50	165.50	189.25	121.00	107.00	55.75	45.75	286.50	296.25
MDD	19	340.00	338.00	16.00	13.50	32.50	37.00	87.52	87.00	63.50	65.00	202.00	197.50	66.50	73.50	71.50	67.00	268.50	271.00
MDD	20	186.00	170.75	91.50	97.50	111.00	120.25	47.88	43.95	182.00	173.00	79.00	66.50	45.50	59.50	61.50	44.75	124.50	126.00
MDD	21	358.75	321.00	2.00	4.25	27.75	63.25	92.34	82.63	69.50	66.00	209.25	162.75	53.75	54.00	95.75	104.25	263.00	216.75
MDD	22	306.25	267.75	21.75	104.50	60.50	16.25	78.83	68.92	153.00	69.75	163.00	139.00	88.50	74.00	54.75	54.75	251.50	213.00
MDD	23	324.00	327.00	48.00	46.00	16.50	15.50	83.40	84.17	235.00	238.50	160.50	179.00	124.50	110.50	39.00	37.50	285.00	289.50
MDD	24	368.50	334.50	4.75	29.75	15.25	24.25	94.85	86.10	122.25	85.25	235.50	207.75	79.75	71.25	53.25	55.50	315.25	279.00
MDD	25	332.75	334.75	20.25	22.75	35.50	31.00	85.65	86.16	120.50	77.25	193.25	149.75	88.00	125.00	51.50	60.00	281.25	274.75
MDD	26	288.00	278.75	69.75	88.75	30.75	21.00	74.13	71.75	145.25	48.75	136.75	184.25	103.50	39.75	47.75	54.75	240.25	224.00
MDD	27	292.50	361.25	13.00	19.50	83.00	7.75	75.29	92.99	85.25	83.50	225.75	230.00	30.50	88.75	36.25	42.50	256.25	318.75
MDD	28	355.00	276.50	2.50	82.00	31.00	30.00	91.38	71.17	145.50	63.50	141.00	166.50	183.50	67.50	30.50	42.50	324.50	234.00
MDD	29	284.25	317.25	12.75	9.00	91.50	62.25	73.17	81.66	167.25	133.25	143.25	160.25	82.75	94.75	58.25	62.25	226.00	255.00
MDD	30	306.25	311.25	49.50	53.25	32.75	24.00	78.83	80.12	97.50	92.25	194.50	182.00	48.25	67.50	63.50	61.75	242.75	249.50
MDD	31	290.50	344.50	59.75	25.50	38.25	18.50	74.77	88.67	122.50	63.25	189.25	187.00	45.75	64.75	55.50	92.75	235.00	251.75

MDD	32	290.25	317.50	51.50	30.75	46.75	40.25	74.71	81.72	84.50	100.25	128.25	147.00	106.25	103.75	55.75	66.75	234.50	250.75
MDD	33	342.00	321.00	21.50	16.75	25.00	50.75	88.03	82.63	133.50	101.00	203.50	174.00	73.25	76.50	65.25	70.50	276.75	250.50
MDD	34	330.00	325.00	28.25	47.00	30.25	16.50	84.94	83.66	132.00	108.00	201.75	179.00	84.75	86.25	43.50	59.75	286.50	265.25
MDD	35	303.25	284.00	38.25	87.75	47.00	16.75	78.06	73.10	124.75	77.25	181.00	158.75	61.50	78.50	60.75	46.75	242.50	237.25
MDD	36	308.50	326.00	36.25	31.00	43.75	31.50	79.41	83.91	154.75	91.00	203.00	163.75	57.00	82.75	48.50	79.50	260.00	246.50
MDD	37	326.25	337.75	41.50	20.50	20.75	30.25	83.98	86.94	67.50	89.00	172.25	173.25	87.75	97.50	66.25	67.00	260.00	270.75
MDD	38	178.50	232.00	117.00	109.00	93.00	47.50	45.95	59.72	172.00	54.00	125.50	142.00	35.00	50.50	18.00	39.50	160.50	192.50
MDD	39	349.50	363.50	14.75	1.50	24.25	23.50	89.96	93.56	54.00	66.50	188.00	159.75	93.50	121.50	68.00	82.25	281.50	281.25
MDD	40	334.25	337.00	44.25	20.50	10.00	31.00	86.04	86.74	78.00	101.00	167.75	205.00	102.00	57.00	64.50	75.00	269.75	262.00
MDD	41	316.75	316.75	49.25	50.75	22.50	21.00	81.53	81.53	36.00	63.50	116.50	151.75	125.50	93.75	74.75	71.25	242.00	245.50
MDD	42	235.25	253.50	75.25	31.50	78.00	103.50	60.55	65.25	68.25	173.50	96.00	142.25	79.25	46.25	60.00	65.00	175.25	188.50
MDD	43	333.50	351.00	5.00	0.50	50.00	37.00	85.84	90.35	185.00	192.50	241.00	187.00	56.50	117.50	36.00	46.50	297.50	304.50
MDD	44	208.00	293.75	37.75	35.25	142.75	59.50	53.54	75.61	NaN	120.25	138.25	190.75	63.25	72.50	6.50	30.50	201.50	263.25
MDD	45	185.25	278.25	95.00	16.75	108.25	93.50	47.68	71.62	81.75	119.25	150.25	192.75	3.00	40.50	32.00	45.00	153.25	233.25
MDD	46	265.00	272.00	43.25	31.75	80.25	84.75	68.21	70.01	190.25	244.75	168.75	215.50	50.50	28.75	45.75	27.75	219.25	244.25
MDD	47	305.00	320.25	41.00	34.00	42.50	34.25	78.51	82.43	76.50	60.50	165.75	184.50	89.50	59.00	49.75	76.75	255.25	243.50
MDD	48	282.25	299.50	48.00	44.00	58.25	45.00	72.65	77.09	37.25	107.00	166.75	156.00	63.75	88.50	51.75	55.00	230.50	244.50
MDD	49	297.25	232.00	44.25	101.25	47.00	55.25	76.51	59.72	125.75	69.00	122.75	91.50	98.00	76.50	76.50	64.00	220.75	168.00
MDD	50	268.25	294.75	21.75	31.25	98.50	62.50	69.05	75.87	122.50	155.50	166.25	189.00	43.50	60.25	58.50	45.50	209.75	249.25
MDD	51	333.00	325.75	46.25	50.25	9.25	12.50	85.71	83.85	66.50	57.00	162.25	160.25	115.25	94.75	55.50	70.75	277.50	255.00
MDD	52	287.75	306.00	27.00	44.75	73.75	37.75	74.07	78.76	147.75	121.50	173.25	211.50	95.25	61.00	19.25	33.50	268.50	272.50
MDD	53	309.75	321.75	13.75	19.50	65.00	47.25	79.73	82.82	162.00	112.75	162.25	206.00	94.00	76.75	53.50	39.00	256.25	282.75
MDD	54	198.50	250.00	73.25	24.25	116.75	114.25	51.09	64.35	NaN	194.50	140.75	165.75	30.00	41.00	27.75	43.25	170.75	206.75
MDD	55	290.50	238.50	19.00	25.00	79.00	125.00	74.77	61.39	73.50	126.50	201.50	149.00	31.50	37.00	57.50	52.50	233.00	186.00
MDD	56	288.00	329.50	16.75	9.75	83.75	49.25	74.13	84.81	77.75	52.50	191.00	218.25	44.50	41.00	52.50	70.25	235.50	259.25
MDD	57	341.50	366.75	22.00	13.00	25.00	8.75	87.90	94.40	71.75	73.25	236.25	206.00	15.75	78.25	89.50	82.50	252.00	284.25
MDD	58	311.50	353.00	16.50	14.50	60.50	21.00	80.18	90.86	39.00	62.00	221.50	184.50	23.50	88.50	66.50	80.00	245.00	273.00
MDD	59	285.50	350.00	30.50	5.50	72.50	33.00	73.49	90.09	90.50	45.00	187.00	259.00	50.00	23.00	48.50	68.00	237.00	282.00
MDD	60	238.00	334.00	55.75	12.25	94.75	42.25	61.26	85.97	87.75	123.25	124.00	177.75	87.50	113.00	26.50	43.25	211.50	290.75
MDD	61	325.00	326.50	30.50	42.00	33.00	20.00	83.66	84.04	109.50	110.00	179.00	222.00	80.50	34.00	65.50	70.50	259.50	256.00
MDD	62	339.25	354.75	19.75	13.25	29.50	20.50	87.32	91.31	82.00	63.00	190.75	204.50	72.00	83.25	76.50	67.00	262.75	287.75
MDD	63	252.50	197.50	60.50	74.50	75.50	116.50	64.99	50.84	162.25	147.00	171.25	138.25	31.50	30.75	49.75	28.50	202.75	169.00
MDD	64	366.50	335.00	15.25	9.75	6.75	43.75	94.34	86.23	75.50	80.75	169.25	200.50	117.00	47.50	80.25	87.00	286.25	248.00

MDD	65	213.50	229.00	63.50	9.00	111.50	150.50	54.95	58.94	51.50	100.50	101.00	129.50	79.50	66.00	33.00	33.50	180.50	195.50
MDD	66	138.00	202.00	43.00	43.25	207.50	143.25	35.52	51.99	41.00	85.50	46.25	124.75	70.00	41.00	21.75	36.25	116.25	165.75
MDD	67	327.00	375.50	35.50	2.50	26.00	10.50	84.17	96.65	192.50	123.00	206.00	223.00	83.00	75.00	38.00	77.50	289.00	298.00
MDD	68	308.50	325.50	20.00	16.50	60.00	46.50	79.41	83.78	75.75	75.25	217.75	188.75	17.00	69.50	73.75	67.25	234.75	258.25
MDD	69	251.75	323.75	56.25	7.75	80.50	57.00	64.80	83.33	84.50	95.50	182.50	211.50	27.25	50.50	42.00	61.75	209.75	262.00
MDD	70	311.50	324.75	14.75	16.75	62.25	47.00	80.18	83.59	69.00	58.50	235.50	180.50	30.50	79.00	45.50	65.25	266.00	259.50
MDD	71	223.75	235.00	62.00	72.75	102.75	80.75	57.59	60.49	NaN	90.00	153.25	140.50	51.50	57.25	19.00	37.25	204.75	197.75
MDD	72	252.75	208.75	21.50	30.25	114.25	149.50	65.06	53.73	82.50	85.75	224.00	147.50	0.00	48.00	28.75	13.25	224.00	195.50
MDD	73	340.00	288.75	27.75	24.25	20.75	75.50	87.52	74.32	107.00	89.75	218.25	179.00	65.25	29.75	56.50	80.00	283.50	208.75
MDD	74	295.25	332.25	2.75	8.50	90.50	47.75	76.00	85.52	71.50	66.75	231.25	232.50	12.25	23.25	51.75	76.50	243.50	255.75
MDD	75	358.00	328.00	11.75	8.25	18.75	52.25	92.15	84.43	115.50	127.50	221.50	168.75	59.75	93.50	76.75	65.75	281.25	262.25
MDD	76	315.75	348.00	12.75	5.75	60.00	34.75	81.27	89.58	59.50	65.00	206.50	218.75	62.75	79.00	46.50	50.25	269.25	297.75
MDD	77	196.50	318.50	24.50	25.50	167.50	44.50	50.58	81.98	264.00	110.50	144.50	209.50	28.50	69.00	23.50	40.00	173.00	278.50
MDD	78	291.50	282.75	29.00	83.00	68.00	22.75	75.03	72.78	133.00	78.25	130.00	104.00	99.50	102.00	62.00	76.75	229.50	206.00
MDD	79	241.00	252.00	56.00	50.00	91.50	86.50	62.03	64.86	70.00	77.50	140.00	140.50	52.00	59.00	49.00	52.50	192.00	199.50
MDD	80	347.00	311.75	15.00	33.00	26.50	43.75	89.32	80.24	48.00	71.75	219.00	219.00	60.50	43.25	67.50	49.50	279.50	262.25
MDD	81	315.50	327.50	12.75	19.50	60.25	41.50	81.21	84.30	121.50	84.25	135.00	158.00	107.00	98.75	73.50	70.75	242.00	256.75
MDD	82	311.00	358.25	38.25	8.75	39.25	21.50	80.05	92.21	57.00	86.75	170.25	199.25	77.75	88.75	63.00	70.25	248.00	288.00
MDD	83	260.00	343.50	22.50	0.50	106.00	44.50	66.92	88.42	NaN	343.00	166.50	277.00	93.50	40.50	0.00	26.00	260.00	317.50
MDD	84	342.75	345.25	20.50	14.25	25.25	29.00	88.22	88.87	230.50	68.00	316.50	183.00	0.25	99.50	26.00	62.75	316.75	282.50
MDD	85	340.50	353.00	26.00	21.00	22.00	14.50	87.64	90.86	167.00	180.50	203.00	172.00	94.50	136.00	43.00	45.00	297.50	308.00
MDD	86	349.00	364.50	26.75	15.50	12.75	8.50	89.83	93.82	163.00	50.75	276.00	195.00	5.75	48.75	67.25	120.75	281.75	243.75
MDD	87	320.00	342.50	57.50	12.50	11.00	33.50	82.37	88.16	123.00	79.50	238.50	251.50	13.50	45.00	68.00	46.00	252.00	296.50
MDD	88	316.00	369.00	46.50	2.00	26.00	17.50	81.34	94.98	121.50	175.50	172.50	168.50	75.00	157.50	68.50	43.00	247.50	326.00
MDD	89	171.50	319.00	102.00	5.00	115.00	64.50	44.14	82.11	151.50	243.50	164.00	216.00	0.00	70.50	7.50	32.50	164.00	286.50
MDD	90	252.25	281.75	15.00	33.25	121.25	73.50	64.93	72.52	51.50	143.50	184.25	177.25	19.75	59.75	48.25	44.75	204.00	237.00
MDD	91	344.00	288.50	10.75	14.75	33.75	85.25	88.55	74.26	65.00	64.75	232.50	175.50	33.75	67.00	77.75	46.00	266.25	242.50
MDD	92	371.50	361.00	8.50	10.50	8.50	17.00	95.62	92.92	80.50	160.50	187.50	193.50	113.50	126.50	70.50	41.00	301.00	320.00
MDD	93	368.50	356.50	9.25	3.25	10.75	28.75	94.85	91.76	109.50	71.50	220.75	198.50	85.50	103.50	62.25	54.50	306.25	302.00
MDD	94	274.75	287.75	38.25	43.00	75.50	57.75	70.72	74.07	211.25	208.00	175.50	195.25	59.75	57.50	39.50	35.00	235.25	252.75
MDD	95	197.50	302.00	172.00	16.00	19.00	70.50	50.84	77.73	19.50	172.50	140.00	205.00	1.00	42.00	56.50	55.00	141.00	247.00
MDD	96	189.00	229.25	54.00	11.00	145.50	148.25	48.65	59.01	203.25	219.25	130.75	123.75	35.75	65.00	22.50	40.50	166.50	188.75
MDD	97	288.50	293.00	96.00	85.50	4.00	10.00	74.26	75.42	172.50	124.50	155.50	181.50	74.50	80.00	58.50	31.50	230.00	261.50

MDD	98	351.00	334.00	29.25	42.50	8.25	12.00	90.35	85.97	62.50	48.25	160.00	162.75	97.50	92.75	93.50	78.50	257.50	255.50
MDD	99	367.00	365.00	15.00	14.00	6.50	9.50	94.47	93.95	71.50	70.00	202.50	179.00	99.00	123.50	65.50	62.50	301.50	302.50
MDD	100	200.00	295.50	56.00	44.00	132.50	49.00	51.48	76.06	255.00	188.00	142.50	203.00	0.50	32.50	57.00	60.00	143.00	235.50
MDD	101	154.00	70.50	208.50	316.50	26.00	1.50	39.64	18.15	122.00	37.00	87.50	36.00	19.00	7.00	47.50	27.50	106.50	43.00
MDD	102	356.00	360.50	11.00	1.50	21.50	26.50	91.63	92.79	28.50	41.50	133.00	221.50	98.50	43.50	124.50	95.50	231.50	265.00
MDD	103	344.50	341.50	8.00	26.00	36.00	21.00	88.67	87.90	119.00	99.50	241.50	222.50	28.50	38.00	74.50	81.00	270.00	260.50
MDD	104	369.50	360.50	15.00	12.00	4.00	16.00	95.11	92.79	145.50	147.00	259.50	208.00	55.00	94.00	55.00	58.50	314.50	302.00
MDD	105	374.75	334.25	5.00	13.50	8.75	40.75	96.46	86.04	137.25	177.00	218.25	167.50	96.75	124.25	59.75	42.50	315.00	291.75
MDD	106	356.00	345.50	7.00	4.00	25.50	39.00	91.63	88.93	104.00	88.00	222.50	189.00	66.00	81.50	67.50	75.00	288.50	270.50
MDD	107	273.50	257.50	75.50	107.00	39.50	24.00	70.40	66.28	91.00	59.00	168.50	118.50	79.00	88.50	26.00	50.50	247.50	207.00
MDD	108	372.50	359.75	3.75	4.75	12.25	24.00	95.88	92.60	69.00	116.25	160.25	172.50	138.50	110.25	73.75	77.00	298.75	282.75
MDD	109	376.50	370.00	4.50	4.25	7.50	14.25	96.91	95.24	66.50	122.50	211.75	181.25	85.25	117.75	79.50	71.00	297.00	299.00
MDD	110	314.25	355.25	32.00	17.50	42.25	15.75	80.89	91.44	89.75	134.50	206.75	180.00	38.50	93.25	69.00	82.00	245.25	273.25
MDD	111	223.25	307.25	72.25	33.25	93.00	48.00	57.46	79.09	NaN	134.50	148.50	199.25	37.25	69.25	37.50	38.75	185.75	268.50
MDD	112	342.00	364.25	5.00	7.00	41.50	17.25	88.03	93.76	105.00	69.75	203.25	282.75	79.00	20.50	59.75	61.00	282.25	303.25
MDD	113	366.00	326.50	14.00	29.75	8.50	32.25	94.21	84.04	90.75	73.75	148.25	134.25	119.50	120.00	98.25	72.25	267.75	254.25
MDD	114	332.50	328.00	14.00	31.00	42.00	29.50	85.59	84.43	50.00	37.50	175.00	209.50	49.00	44.50	108.50	74.00	224.00	254.00
MDD	115	150.50	155.25	183.25	218.50	54.75	14.75	38.74	39.96	66.25	NaN	40.75	87.25	17.75	25.50	92.00	42.50	58.50	112.75
MDD	116	317.25	267.00	31.50	66.00	39.75	55.50	81.66	68.73	76.25	137.00	146.25	144.75	58.00	43.75	113.00	78.50	204.25	188.50
MDD	117	145.00	151.00	226.00	229.75	17.50	7.75	37.32	38.87	75.75	71.00	85.00	102.50	45.75	35.50	14.25	13.00	130.75	138.00
MDD	118	241.50	239.50	115.00	132.00	32.00	17.00	62.16	61.65	NaN	67.00	190.00	132.50	51.50	46.50	0.00	60.50	241.50	179.00
MDD	119	346.00	354.50	25.50	7.50	17.00	26.50	89.06	91.25	NaN	122.00	320.50	227.50	25.50	73.00	0.00	54.00	346.00	300.50
MDD	120	298.25	343.25	11.75	24.75	78.50	20.50	76.77	88.35	78.00	65.50	158.25	169.00	73.00	103.50	67.00	70.75	231.25	272.50
MDD	121	106.00	129.50	99.00	122.50	183.50	136.50	27.28	33.33	231.00	217.00	81.00	91.00	2.00	25.50	23.00	13.00	83.00	116.50
MDD	122	256.50	276.25	100.75	102.75	31.25	9.50	66.02	71.11	38.75	60.00	106.75	186.50	48.25	39.00	101.50	50.75	155.00	225.50
MDD	123	358.50	365.50	12.50	14.00	17.50	9.00	92.28	94.08	99.50	96.50	231.50	228.50	44.00	60.00	83.00	77.00	275.50	288.50
MDD	124	290.50	312.25	48.00	36.75	50.00	39.50	74.77	80.37	105.00	160.25	196.25	139.25	60.00	134.50	34.25	38.50	256.25	273.75
Health	1	310.50	322.00	19.75	14.25	58.25	52.25	79.92	82.88	91.75	181.75	163.00	176.25	64.25	71.00	83.25	74.75	227.25	247.25
Health	2	315.25	356.25	51.50	20.25	21.75	12.00	81.15	91.70	57.25	84.25	175.50	154.50	65.50	105.25	74.25	96.50	241.00	259.75
Health	3	378.00	368.50	3.50	2.50	7.00	17.50	97.30	94.85	59.00	57.50	145.00	141.00	152.50	110.00	80.50	117.50	297.50	251.00
Health	4	379.00	364.00	5.00	9.75	4.50	14.75	97.55	93.69	60.75	50.25	186.75	179.50	145.50	108.00	46.75	76.50	332.25	287.50
Health	5	325.25	301.75	32.25	33.50	31.00	53.25	83.72	77.67	125.00	123.75	181.75	165.00	107.75	82.00	35.75	54.75	289.50	247.00
Health	6	355.00	351.25	13.75	13.50	19.75	23.75	91.38	90.41	54.00	55.75	197.00	187.75	75.00	91.25	83.00	72.25	272.00	279.00

Health	7	375.25	378.00	4.50	2.75	8.75	7.75	96.59	97.30	240.00	62.00	179.25	204.25	181.25	106.75	14.75	67.00	360.50	311.00
Health	8	376.00	352.00	3.00	1.00	9.50	35.50	96.78	90.60	84.50	76.50	103.00	127.50	210.00	153.50	63.00	71.00	313.00	281.00
Health	9	363.25	343.00	5.25	14.50	20.00	31.00	93.50	88.29	66.50	56.25	127.50	182.75	167.50	77.75	68.25	82.50	295.00	260.50
Health	10	349.25	337.25	16.00	17.75	23.25	33.50	89.90	86.81	82.00	77.75	98.50	187.00	215.25	74.00	35.50	76.25	313.75	261.00
Health	11	338.50	337.50	8.50	6.25	41.50	44.75	87.13	86.87	39.75	64.50	191.50	206.25	74.00	68.25	73.00	63.00	265.50	274.50
Health	12	324.50	315.50	10.75	8.75	53.25	64.25	83.53	81.21	108.00	109.00	185.00	180.75	94.50	69.75	45.00	65.00	279.50	250.50
Health	13	354.00	337.75	6.25	12.25	28.25	38.50	91.12	86.94	92.00	85.25	209.75	160.25	74.75	93.75	69.50	83.75	284.50	254.00
Health	14	379.25	373.75	3.50	5.00	5.75	9.75	97.62	96.20	81.75	75.50	123.00	171.50	212.50	120.25	43.75	82.00	335.50	291.75
Health	15	351.75	344.13	3.38	3.25	33.38	41.13	90.54	88.58	39.50	40.88	247.13	160.50	23.75	100.00	80.88	83.63	270.88	260.50
Health	16	338.50	297.00	8.20	7.90	41.80	83.60	87.13	76.45	72.10	71.90	181.80	126.50	78.70	101.20	78.00	69.30	260.50	227.70
Health	17	366.50	363.10	7.90	7.80	14.10	17.60	94.34	93.46	106.10	107.70	226.50	164.40	80.10	132.20	59.90	66.50	306.60	296.60
Health	18	366.17	368.00	6.50	5.67	15.83	14.83	94.25	94.72	74.67	72.33	199.00	186.50	74.67	89.33	92.50	92.17	273.67	275.83
Health	19	362.50	347.60	12.30	23.40	13.70	17.50	93.31	89.47	69.30	80.70	221.80	193.90	68.40	80.30	72.30	73.40	290.20	274.20
Health	20	360.80	344.30	14.80	18.50	12.90	25.70	92.87	88.62	62.70	79.00	198.20	174.60	76.50	99.20	86.10	70.50	274.70	273.80
Health	21	363.50	366.00	10.40	8.30	14.60	14.20	93.56	94.21	49.90	61.30	219.10	201.30	63.60	82.60	80.80	82.10	282.70	283.90
Health	22	341.60	334.80	5.20	8.10	41.70	45.60	87.93	86.18	78.20	92.90	206.60	193.30	64.00	70.40	71.00	71.10	270.60	263.70
Health	23	381.50	379.50	5.50	2.50	1.50	6.50	98.20	97.68	53.00	56.50	235.50	220.50	66.00	84.50	80.00	74.50	301.50	305.00
Health	24	368.20	363.60	11.00	13.40	9.30	11.50	94.77	93.59	59.70	68.30	216.10	162.00	57.80	96.40	94.30	105.20	273.90	258.40
Health	25	338.90	343.50	14.90	17.70	34.70	27.30	87.23	88.42	127.70	123.20	251.70	219.30	25.00	72.00	62.20	52.20	276.70	291.30
Health	26	366.70	347.90	13.30	8.80	8.50	31.80	94.39	89.55	63.90	87.40	189.90	183.20	93.80	93.10	83.00	71.60	283.70	276.30
OSA	1	247.50	148.25	13.50	43.25	127.50	197.00	63.71	38.16	185.25	238.75	196.25	103.25	22.75	7.75	28.50	37.25	219.00	111.00
OSA	2	356.00	343.50	7.00	15.50	25.50	29.50	91.63	88.42	51.00	41.00	169.00	176.00	117.00	86.00	70.00	81.50	286.00	262.00
OSA	3	252.50	240.50	70.00	68.00	66.00	80.00	64.99	61.90	165.50	161.00	174.50	115.00	35.00	91.50	43.00	34.00	209.50	206.50
OSA	4	342.50	340.50	23.00	22.50	23.00	25.50	88.16	87.64	39.50	95.50	207.50	165.00	65.50	107.50	69.50	68.00	273.00	272.50
OSA	5	366.50	353.50	4.50	15.50	17.50	19.50	94.34	90.99	97.50	82.50	202.00	173.50	119.50	95.00	45.00	85.00	321.50	268.50
OSA	6	289.50	356.50	35.50	8.00	63.50	24.00	74.52	91.76	66.00	91.50	239.00	265.00	5.50	28.50	45.00	63.00	244.50	293.50
OSA	7	282.50	281.50	13.50	1.50	92.50	105.50	72.72	72.46	126.00	99.50	233.00	176.00	0.00	56.00	49.50	49.50	233.00	232.00
OSA	8	216.00	247.50	35.50	25.00	137.00	116.00	55.60	63.71	63.50	75.50	125.50	119.50	49.50	72.50	41.00	55.50	175.00	192.00
OSA	9	285.50	307.50	25.50	33.00	77.50	48.00	73.49	79.15	141.00	129.50	183.00	130.00	42.50	102.00	60.00	75.50	225.50	232.00
OSA	10	313.00	330.50	17.50	9.50	58.00	48.50	80.57	85.07	189.00	54.50	237.50	179.00	33.00	61.50	42.50	90.00	270.50	240.50
OSA	11	341.00	322.00	9.50	9.00	38.00	57.50	87.77	82.88	53.50	49.50	158.50	142.00	102.50	112.50	80.00	67.50	261.00	254.50
OSA	12	330.50	214.00	19.50	144.50	38.50	30.00	85.07	55.08	17.50	51.50	63.50	100.00	219.00	67.00	48.00	47.00	282.50	167.00
OSA	13	372.00	354.50	2.50	9.50	14.00	24.50	95.75	91.25	135.00	130.00	282.50	216.00	9.50	89.00	80.00	49.50	292.00	305.00

OSA	14	328.75	332.00	19.25	12.00	40.50	44.50	84.62	85.46	109.50	57.50	167.50	163.75	119.50	88.75	41.75	79.50	287.00	252.50
OSA	15	337.00	348.50	28.00	26.50	23.50	13.50	86.74	89.70	128.50	51.50	206.00	198.00	79.00	83.50	52.00	67.00	285.00	281.50
OSA	16	266.00	310.50	93.00	32.00	29.50	46.00	68.47	79.92	67.00	49.50	169.50	187.50	67.00	64.00	29.50	59.00	236.50	251.50
OSA	17	290.00	325.50	83.00	11.50	15.50	51.50	74.65	83.78	64.50	46.00	174.00	226.50	70.00	45.50	46.00	53.50	244.00	272.00
OSA	18	360.50	348.50	3.00	10.50	25.00	29.50	92.79	89.70	86.50	77.00	223.00	182.00	64.00	88.00	73.50	78.50	287.00	270.00
OSA	19	332.50	320.50	32.50	27.00	23.50	41.00	85.59	82.50	115.00	85.50	207.50	141.00	71.00	87.50	54.00	92.00	278.50	228.50
OSA	20	302.50	276.00	14.50	48.50	71.50	64.00	77.86	71.04	103.50	75.00	205.00	171.50	63.00	64.00	34.50	40.50	268.00	235.50
OSA	21	161.00	329.50	151.50	15.00	76.00	44.00	41.44	84.81	89.50	216.00	158.50	232.50	0.00	69.50	2.50	27.50	158.50	302.00
OSA	22	285.50	159.50	10.00	82.50	93.00	146.50	73.49	41.06	108.50	111.50	190.50	132.00	14.50	6.00	80.50	21.50	205.00	138.00
OSA	23	252.50	243.00	11.50	13.50	124.50	132.00	64.99	62.55	89.50	159.00	135.00	215.00	74.00	0.00	43.50	28.00	209.00	215.00
OSA	24	221.50	297.50	32.00	24.00	135.00	67.00	57.01	76.58	256.00	145.00	173.50	184.50	25.50	84.50	22.50	28.50	199.00	269.00
OSA	25	200.50	278.00	13.00	20.50	175.00	90.00	51.61	71.56	291.00	293.50	197.00	199.50	0.00	42.00	3.50	36.50	197.00	241.50
OSA	26	344.00	345.50	17.00	9.00	27.50	34.00	88.55	88.93	50.50	190.00	229.50	246.50	56.00	69.00	58.50	30.00	285.50	315.50
OSA	27	287.50	339.00	4.00	0.50	97.00	49.00	74.00	87.26	64.50	53.00	238.50	248.50	16.50	48.00	32.50	42.50	255.00	296.50
OSA	28	293.50	265.00	15.50	86.50	79.50	37.00	75.55	68.21	138.50	54.50	241.00	149.00	31.00	34.00	21.50	82.00	272.00	183.00

Legend

Patho: Pathlogy groups
INS: Insomnia
MDD: Major depressive disorder
OSA: Obstructive sleep apnea

Subj: Subjects ID

PSG: Polysomnography
SA: Somno-Art Software

Sleep parameters:

TST: total sleep time
SE: Sleep efficiency
SL: Sleep latency
WASO: Wake after sleep onset
REML: REM sleep latency

N1N2: N1+N2 sleep
N3: N3 sleep
REM: REM sleep
NREM: NREM sleep
