# Supplementary material for: Somno-Art Software identifies pathology-induced changes in sleep parameters similarly to polysomnography
Source: PLoS One. 2023 Oct 20;18(10):e0291593. doi: 10.1371/journal.pone.0291593 (PMC10588897; doi:10.1371/journal.pone.0291593)
Supplement: Attachment — Submitted filename: Response to reviewers.docx [file pone.0291593.s003.docx]

Reviewers' comments:

Reviewer's Responses to Questions

**Comments to the Author**

1. Is the manuscript technically sound, and do the data support the conclusions?

The manuscript must describe a technically sound piece of scientific research with data that supports the conclusions. Experiments must have been conducted rigorously, with appropriate controls, replication, and sample sizes. The conclusions must be drawn appropriately based on the data presented.

Reviewer #1: Yes

Reviewer #2: Yes

2. Has the statistical analysis been performed appropriately and rigorously?

Reviewer #1: No

Reviewer #2: Yes

3. Have the authors made all data underlying the findings in their manuscript fully available?

The [PLOS Data policy](http://www.plosone.org/static/policies.action#sharing) requires authors to make all data underlying the findings described in their manuscript fully available without restriction, with rare exception (please refer to the Data Availability Statement in the manuscript PDF file). The data should be provided as part of the manuscript or its supporting information, or deposited to a public repository. For example, in addition to summary statistics, the data points behind means, medians and variance measures should be available. If there are restrictions on publicly sharing data—e.g. participant privacy or use of data from a third party—those must be specified.

Reviewer #1: Yes

Reviewer #2: Yes

4. Is the manuscript presented in an intelligible fashion and written in standard English?

PLOS ONE does not copyedit accepted manuscripts, so the language in submitted articles must be clear, correct, and unambiguous. Any typographical or grammatical errors should be corrected at revision, so please note any specific errors here.

Reviewer #1: Yes

Reviewer #2: Yes

5. Review Comments to the Author

Please use the space provided to explain your answers to the questions above. You may also include additional comments for the author, including concerns about dual publication, research ethics, or publication ethics. (Please upload your review as an attachment if it exceeds 20,000 characters)

Reviewer #1: The main problem of the manuscript is a poor statistical analysis. From my point of view, the authors should analyse sleep parameters using a mixed model ANOVA taking into account the technique of sleep evaluation (PSG vs. SomnoArt) and the clinical status (Healthy subjects vs. OSA, Insomnia, and MDD patients respectively) rather than multiple comparisons using Mann-Whitney U-test. The absence of significant 'technique' effect and significant 'technique x clinical status' interactions would be more demonstrative results.
The results section and the discussion must therefore be rewritten and a synthesis of the results should also be presented in one or two tables.

**Answer:** Thank you for your review. We understand your concern with the statistical analysis. We decided not to include the mixed model analysis, as the goal of this publication was to check if Somno-Art can detect changes in sleep parameters observed in sleep disorders compared to healthy. Comparing the performances of Somno-Art to PSG in healthy subjects and pathologies, has already been published in previous publication.

However, as you suggested, we integrated the mixed model analysis to give an overview. The fact that the interactions ‘group x technology’  were non-significant suggests that the differences in pathologies are independent of the technology used. In a second step we kept the Mann-Whitney U-tests to have an in-depth analysis of the differences we are interested in (healthy vs OSA, healthy vs INS, healthy vs MDD).

Reviewer #2: This is an interesting paper which highlighted the possibilities of using Somno-Art in the relevant setting. A concise and useful paper indicating that there may indeed be a place for Somno-Art in such a setting.

**Answer:** We thank reviewer 2 for the review.

6. PLOS authors have the option to publish the peer review history of their article ([what does this mean?](https://journals.plos.org/plosone/s/editorial-and-peer-review-process#loc-peer-review-history)). If published, this will include your full peer review and any attached files.

If you choose “no”, your identity will remain anonymous but your review may still be made public.


**Do you want your identity to be public for this peer review?** For information about this choice, including consent withdrawal, please see our [Privacy Policy](https://www.plos.org/privacy-policy).

Reviewer #1: **Yes:** Dr Olivier COSTE (Lyon), MD-PhD

Reviewer #2: No
